# What Does DALL-E 2 Know About Radiology?

**DOI:** 10.2196/43110

**Published:** 2023-03-16

**Authors:** Lisa C Adams, Felix Busch, Daniel Truhn, Marcus R Makowski, Hugo J W L Aerts, Keno K Bressem

**Affiliations:** 1 Department of Radiology Charité Universitätsmedizin Berlin Corporate Member of Freie Universität Berlin and Humboldt Universität zu Berlin Berlin Germany; 2 Department of Radiology Stanford University Stanford, CA United States; 3 Department of Diagnostic and Interventional Radiology University Hospital Aachen Aachen Germany; 4 Department of Diagnostic and Interventional Radiology Technical University of Munich Munich Germany; 5 Artificial Intelligence in Medicine Program (AIM) Mass General Brigham Harvard Medical School Boston, MA United States; 6 Radiology and Nuclear Medicine, CARIM & GROW Maastricht University Maastricht Netherlands

**Keywords:** DALL-E, creating images from text, image creation, image generation, transformer language model, machine learning, generative model, radiology, x-ray, artificial intelligence, medical imaging, text-to-image, diagnostic imaging

## Abstract

Generative models, such as DALL-E 2 (OpenAI), could represent promising future tools for image generation, augmentation, and manipulation for artificial intelligence research in radiology, provided that these models have sufficient medical domain knowledge. Herein, we show that DALL-E 2 has learned relevant representations of x-ray images, with promising capabilities in terms of zero-shot text-to-image generation of new images, the continuation of an image beyond its original boundaries, and the removal of elements; however, its capabilities for the generation of images with pathological abnormalities (eg, tumors, fractures, and inflammation) or computed tomography, magnetic resonance imaging, or ultrasound images are still limited. The use of generative models for augmenting and generating radiological data thus seems feasible, even if the further fine-tuning and adaptation of these models to their respective domains are required first.

## The Potential Impact of Generative Models in Radiology

Artificial intelligence is often seen as a potential transformer of medicine [1]. DALL-E 2 is a novel deep learning model for text-to-image generation that was first introduced by OpenAI in April 2022 [2]. The model has recently gained widespread public interest due to its ability to create photorealistic images solely from short written inputs [3-5]. Trained on billions of text-image pairs extracted from the internet, DALL-E 2 learned a wide range of representations that it can recombine to create novel images, thereby exceeding the variability it has seen in the training data, even when given implausible prompts (eg, “a painting of a pelvic X-ray by Claude Monet”) [2,6-8].

DALL-E 2’s powerful generative capabilities raise the question of whether these can be transferred to the medical domain to create or augment data, as medical data can be sparse and hard to come by [9,10]. Given the large number of images on which DALL-E 2 was trained, it is reasonable to assume that the training data for DALL-E 2 included radiological images and that the model may have learned something about the composition and structure of x-ray, cross-sectional, and ultrasound images.

This viewpoint article describes how we systematically examined the radiological knowledge embedded in DALL-E 2 by creating and manipulating x-ray, computed tomography (CT), magnetic resonance imaging (MRI), and ultrasound images.

## Generating Radiological Images From Short Descriptive Texts

**Figure 1:**
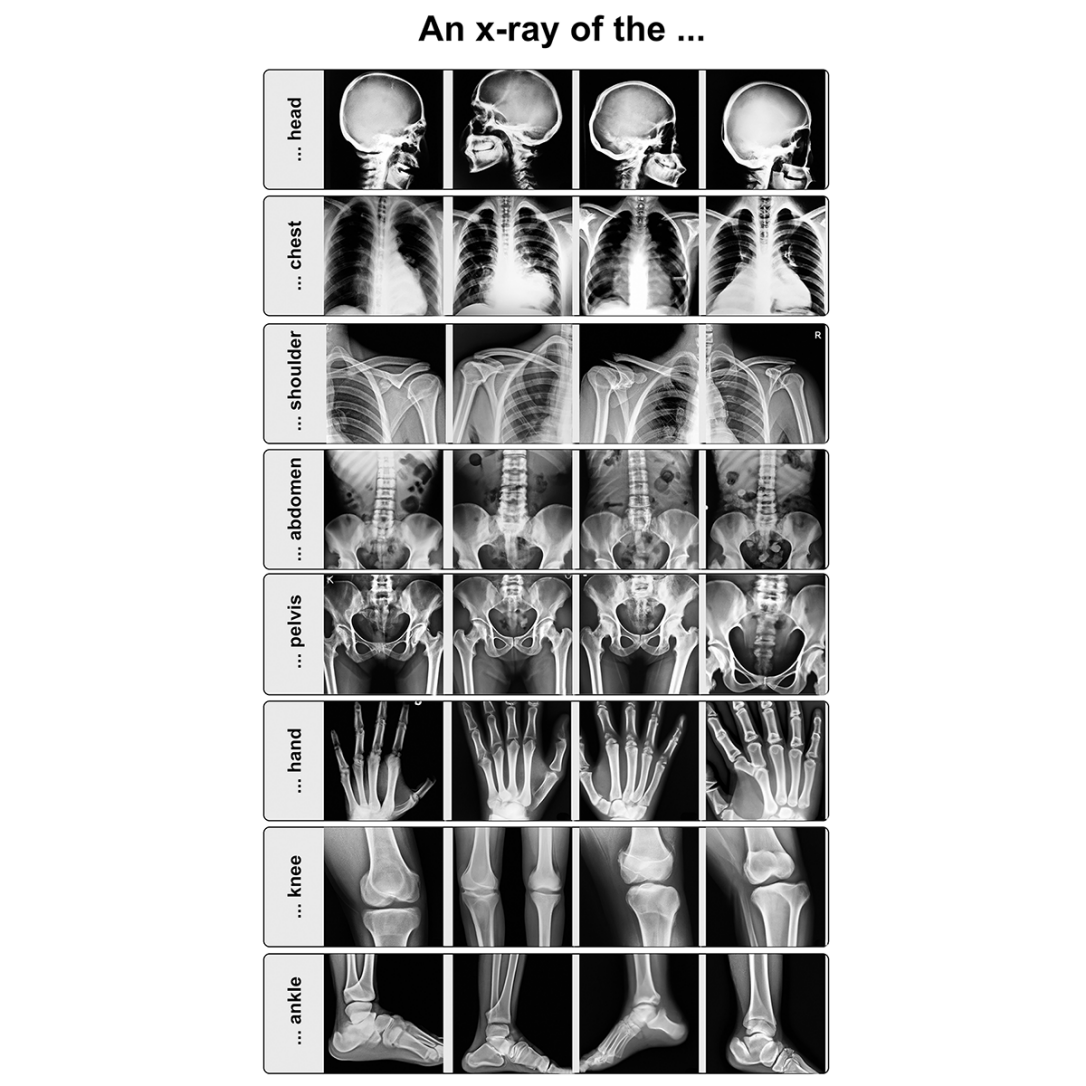
Examples of anatomical structures in x-ray images that were created with DALL-E 2 based on short text descriptions.

To investigate the general radiological domain knowledge embedded in DALL-E 2, we first tested how well radiological images could be created from short descriptive texts by using the words “An X-ray of” and a 1-word description of an anatomical area. For the head, chest, shoulder, abdomen, pelvis, hand, knee, and ankle, we let DALL-E 2 synthesize 4 artificial x-ray images, which are displayed in Figure 1. For each prompt, the model produced 4 outputs, of which all are presented in order to avoid selection bias.

The coloring of the images and the general structure of the bones appeared realistic, and the overall anatomical propositions were correct, indicating the presence of fundamental concepts of x-ray anatomy. However, upon close inspection, it was noted that the trabecular structure of the bone appeared random and did not follow the course of mechanical stress as it would in real x-rays. Sometimes, smaller bones were missing, like the fibula in multiple x-rays of the knee, or multiple small bones, such as several carpal or tarsal bones, were fused into 1 bone. Nevertheless, the overall structure of the depicted anatomic area was recognizable. In rare cases, additional bones and joints were created, such as an additional index finger in a hand x-ray. The model had the greatest difficulties in generating joints correctly, as joints appeared to be fused, or the joint surfaces were distorted. Finally, the quality of the images was not what one would expect for clinic x-ray images. The collimation was largely not correct, with parts of the organ missing or the relevant regions not being centered. Examples of this are visible in Figure 1, wherein the tips of the fingers are missing in all hand x-rays, and the outer parts of the pelvis are missing in one image.

**Figure 2:**
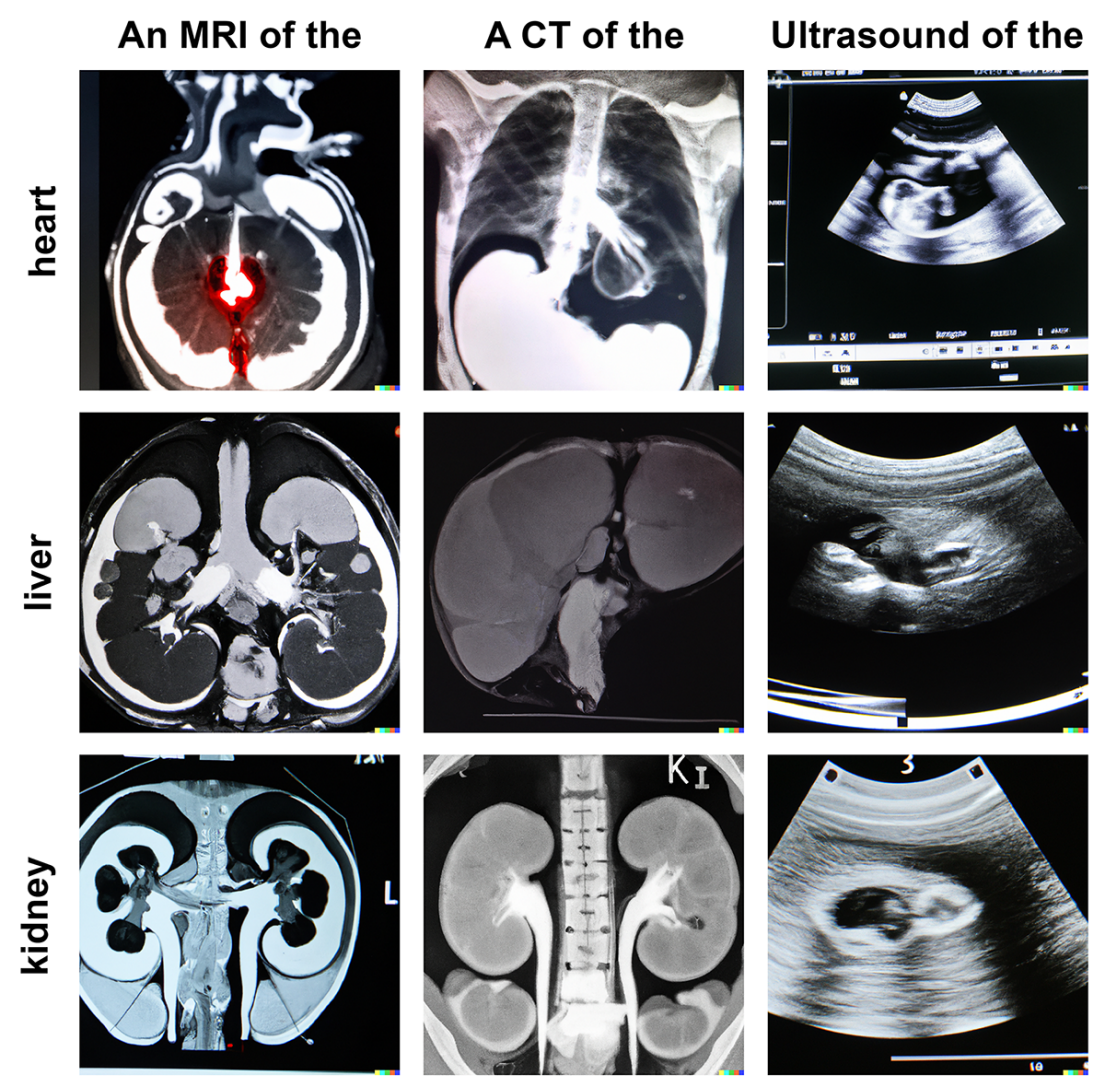
Examples of text-to-image–generated anatomical structures in CT, MRI, and ultrasound images created with DALL-E 2. CT: computed tomography; MRI: magnetic resonance imaging.

When compared to x-rays, the generation of cross-sectional images from CT or MRI was very limited (Figure 2). Although the desired anatomical structure was discernible on the images, the overall impression was predominantly nonsensical. Instead of a cross-sectional image, a photograph of an entire CT or MRI examination on x-ray film was often shown. On ultrasound images, the desired anatomic structures were not discernible; instead, all images resembled obstetric images. Nevertheless, the images showed concepts of CT, MRI, and ultrasound images, suggesting that representations of these modalities are present in DALL-E 2.

## Changing Areas in Radiological Images While Maintaining the Anatomical Structure

**Figure 3:**
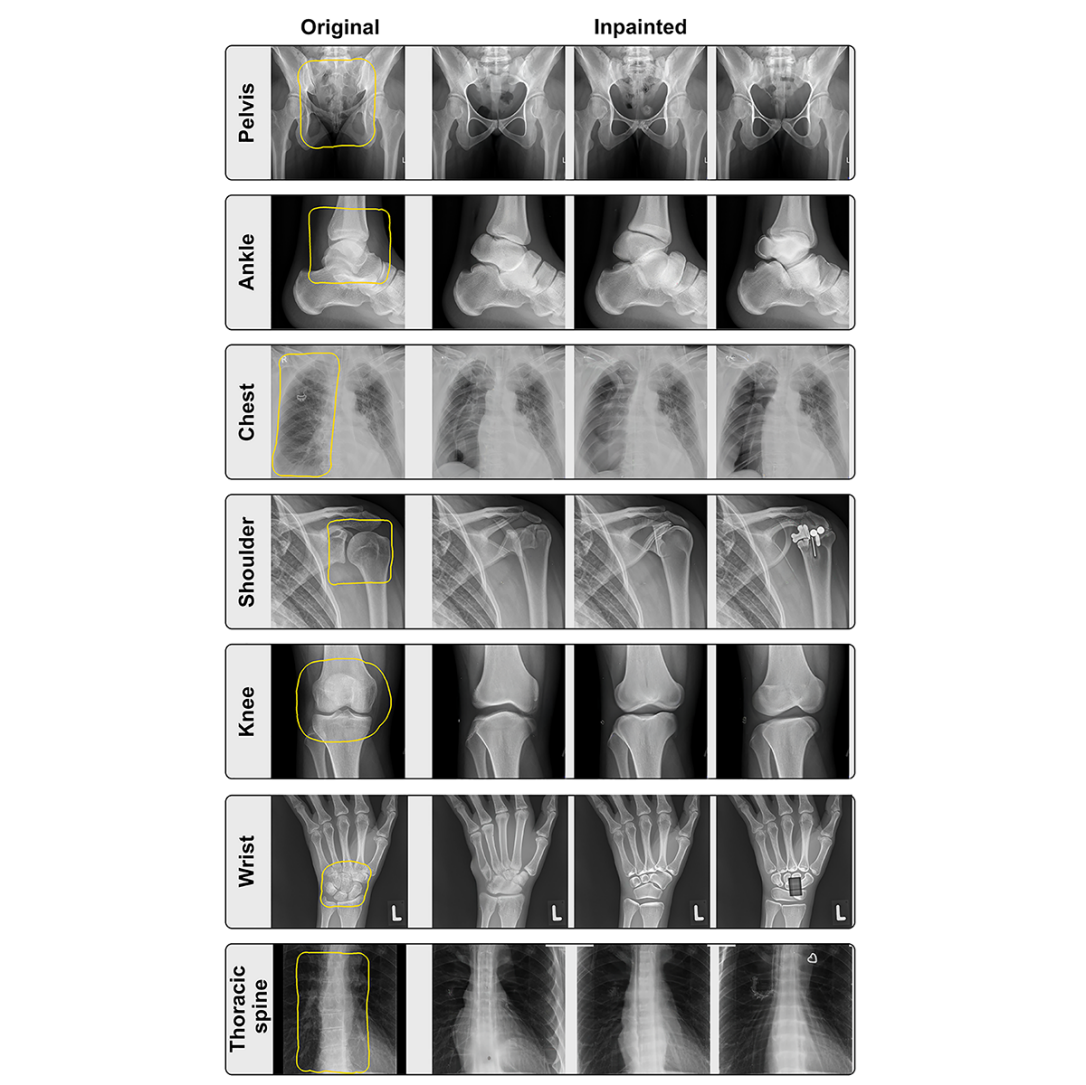
Reconstructed areas of different anatomical locations in x-rays created by using DALL-E 2. The yellow-bordered regions of the original images were erased before providing the remnant images for reconstruction.

To investigate the extent of DALL-E 2’s radiological knowledge, we tested how well the modal can reconstruct missing areas in a radiological image (inpainting). To do this, we selected radiographs of the pelvis, ankle, chest, shoulder, knee, wrist, and thoracic spine and erased specific areas before handing the remnants to DALL-E 2 for reconstruction (Figure 3). The accompanying prompts were identical to the those used for generating the text-based images described in the *Generating Radiological Images From Short Descriptive Texts* section. DALL-E 2 provided realistic replacement images that were nearly indistinguishable from the original radiographs of the pelvis, thorax, and thoracic spine. However, the results were not as convincing when a joint was included in the image area. In the ankle and wrist images, the number of tarsal bones and the structure varied greatly from those in the original radiographs and deviated from realistic representations. For the shoulder images, DALL-E 2 failed to reconstruct the glenoid cavity and articular surface of the humerus. In one image, a foreign body was inserted into the shoulder that remotely resembled a prosthesis. Further, when reconstructing the knee image, the model omitted the patella but retained the bicondylar structure of the femur.

## Extending Radiological Images Beyond Image Borders

**Figure 4:**
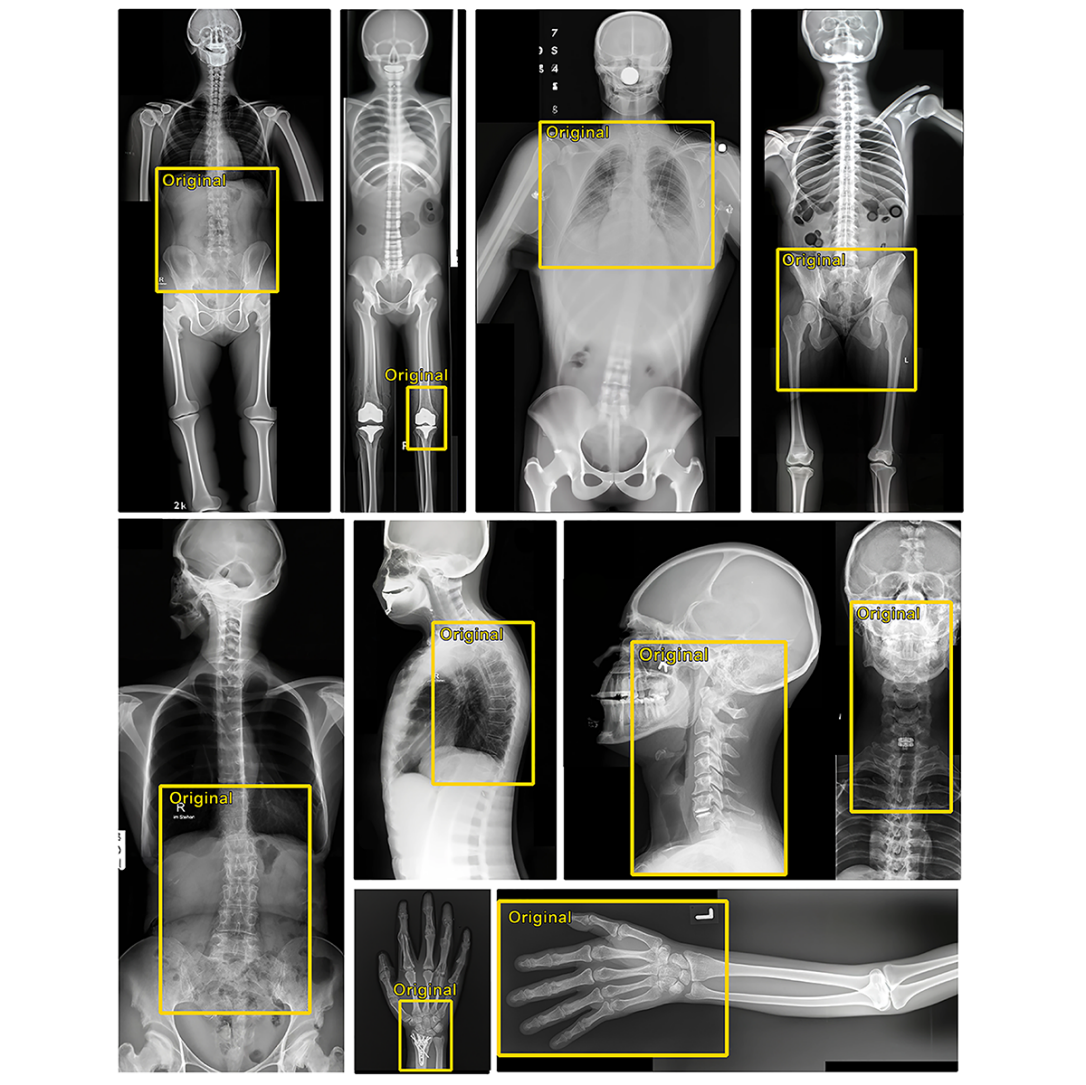
Extending x-ray images of different anatomical regions beyond their borders by using DALL-E 2. The original x-rays are shown in yellow boxes, and the areas outside of the yellow boxes were generated by DALL-E 2.

Since the previous two experiments showed that DALL-E 2 could handle the basics of standard radiological images, we also wanted to investigate how well anatomical knowledge is embedded in the model. To do this, we randomly selected radiographic images of the abdomen, chest, pelvis, knee, various spinal regions, wrist bones, and hand and had DALL-E 2 extend these images beyond their boundaries, since the model needs to know both locations and anatomical proportions for this task. The accompanying prompts were selected based on the anatomical regions to be created. Again, the style of the augmented images represented a realistic representation of radiographs (Figure 4). It was possible to create a complete full-body radiograph by using only 1 image of the knee as a starting point. The greater the distance between the original image and the generated area, the less detailed the images became. Anatomical proportions, such as the length of the femur or the size of the lung, remained realistic, but finer details, such as the number of lumbar vertebrae, were inconsistent. The model generally performed best when creating anterior and posterior views, while the creation of lateral views was more challenging and produced poorer results.

## Generation of Pathological Images

The generation of pathological images to, for example, visualize fractures, intracranial hemorrhages, or tumors was limited. We tested this by generating fractures on radiographs, which mostly resulted in distorted images that were similar to those in Figure 1. In addition, DALL-E 2 has a filter that prevents the generation of harmful content. Therefore, we could not test most pathologies because words such as “bleeding” triggered this filter.

## Conclusion and Outlook

We were able to show that DALL-E 2 can generate x-ray images that are similar to authentic x-ray images in terms of style and anatomical proportions. Thus, we conclude that relevant representations for x-rays were learned by DALL-E 2 during training. However, it seems that only representations for radiographs without pathologies are available or are allowed by the filter, as the generation of pathological images (eg, images of fractures and tumors) was limited. In addition, DALL-E 2’s generative capabilities were poor for CT, MRI, and ultrasound images.

Access to data is critical in deep learning, and in general, the larger the data set, the better the performance of the models trained on the data set. However, especially in radiology, there is no single large database from which to create such a data set. Instead, the necessary data are divided among several institutions, and privacy concerns additionally prevent the merging of these data. Synthetic data from generative models, such as DALL-E 2, show promise for addressing these issues by enabling the creation of data sets that are much larger than those that are currently available and greatly accelerating the development of new deep learning tools for radiology [11,12].

Previous approaches to data generation via generative adversarial networks were hampered by instabilities during training and the inability to capture sufficient scatter in the training data [13,14]. In contrast, novel diffusion-based generative models, of which DALL-E 2 is a prominent representative, solved these problems and outperformed generative adversarial networks in image generation [15].

Diffusion models are very interesting for augmenting radiological data due to their control capabilities and flexibility in data generation, as images can be modified, and pathologies can be added or removed simply by using text inputs. Nevertheless, generated images should be subjected to quality control by domain experts to reduce the risk of incorrect information from DALL-E 2 entering a generated data set. This would help to avoid possible negative effects on models trained with these data or on individuals using these data for training.

The biggest impact of these text-to-image models in radiology could be data generation for research and education. Notably, our experiments showed that DALL-E 2, in its current form, was not able to produce convincing pathological images, probably due to the lack of such images in the training data. Radiological images containing a variety of pathologies are more likely to be provided on professional websites, may be under restricted access, or are stored as specialized data formats (eg, DICOM). Overall, these restrictions likely resulted in only a small number of images being included in the training data. In addition, the images in the data could be distorted. When looking at Figures 1 and 4, the absence of breast shadows is noticeable, leading to the assumption that all images probably show men, which makes gender bias in the model likely. Furthermore, because there is no way to ensure that the images in the training data come only from curated medical websites, incorrect information in these images may cause the model to learn incorrect concepts and thus produce biased results.

Further work is needed to explore diffusion models for medical data generation and modification [16]. Although DALL-E 2 is not publicly available, other models with a similar architecture, such as Stable Diffusion (Stability AI), were recently made available to the public [17].

Future research should focus on fine-tuning these models to medical data and incorporating medical terminology to create powerful models for data generation and augmentation in radiology research.

## Abbreviations

CT: computed tomography
MRI: magnetic resonance imaging

## Data Availability

All generated research data are included in the manuscript.
